# Point-of-Care Diagnostic Test for Beta-Thalassemia

**DOI:** 10.3390/bios14020083

**Published:** 2024-02-02

**Authors:** Ran An, Alireza Avanaki, Priyaleela Thota, Sai Nemade, Amrish Mehta, Umut A. Gurkan

**Affiliations:** 1 Department of Mechanical and Aerospace Engineering, Case Western Reserve University, Cleveland, OH 44106, USA; 2 Department of Biomedical Engineering, University of Houston, Houston, TX 77004, USA; 3 Department of Biomedical Sciences, University of Houston, Houston, TX 77004, USA; 4 HemexHealth, Inc., Portland, OR 97239, USAp.thota@hemexhealth.com (P.T.); 5 Plasma Lab, Jalgaon 425001, Indiadramrishmehta@gmail.com (A.M.); 6 Apple Diagnostics Lab, Ghatkopar, Mumbai 400077, India; 7 Department of Biomedical Engineering, Case Western Reserve University, Cleveland, OH 44106, USA; 8 Case Comprehensive Cancer Center, Case Western Reserve University, Cleveland, OH 44106, USA

**Keywords:** hemoglobin variants, hemoglobin disorders, *β*-thalassemia, anemia, point-of-care screening, microchip electrophoresis

## Abstract

Hemoglobin (Hb) disorders are among the most common monogenic diseases affecting nearly 7% of the world population. Among various Hb disorders, approximately 1.5% of the world population carries *β*-thalassemia (*β*-Thal), affecting 40,000 newborns every year. Early screening and a timely diagnosis are essential for *β*-thalassemia patients for the prevention and management of later clinical complications. However, in Africa, Southern Europe, the Middle East, and Southeast Asia, where *β*-thalassemia is most prevalent, the diagnosis and screening for *β*-thalassemia are still challenging due to the cost and logistical burden of laboratory diagnostic tests. Here, we present Gazelle, which is a paper-based microchip electrophoresis platform that enables the first point-of-care diagnostic test for *β*-thalassemia. We evaluated the accuracy of Gazelle for the *β*-Thal screening across 372 subjects in the age range of 4–63 years at Apple Diagnostics lab in Mumbai, India. Additionally, 30 blood samples were prepared to mimic *β*-Thal intermediate and *β*-Thal major samples. Gazelle-detected levels of Hb A, Hb F, and Hb A_2_ demonstrated high levels of correlation with the results reported through laboratory gold standard high-performance liquid chromatography (HPLC), yielding a Pearson correlation coefficient = 0.99. This ability to obtain rapid and accurate results suggests that Gazelle may be suitable for the large-scale screening and diagnosis of *β*-Thal.

## 1. Introduction

Hemoglobin (Hb) disorders are among the world’s most common monogenic diseases [1]. *β*-thalassemia (*β*-Thal) is caused by single mutations resulting in small deletions or insertions within either the *β*-*globin* gene or its immediate flanking sequence or by gross deletions resulting in a reduced production of normal hemoglobin (Hb A). Globally, approximately 1.5% of the world population carries *β*-Thal, which affects 40,000 newborns every year [2,3]. It is estimated that over 90% of patients with *β*-Thal live in low-and middle-resource settings, including those found in Africa, the Middle East, Southeast Asia, and Southern Europe [4].

The early detection and screening for *β*-Thal are crucial for enabling timely interventions [5], preventing complications [5], facilitating informed family planning through genetic counseling [6], supporting public health strategies in high-prevalence areas [7], improving patients’ quality of life [8], and enhancing education and awareness [9]. Successful screening and prevention programs have reduced the number of persons affected by *β*-Thal in countries such as Cyprus, Greece, and Italy [10]. However, such programs have not been widely implemented in other areas due in large part to the cost and logistical burden of laboratory diagnostic tests [10].

There are many *β*-Thal syndromes. The most common ones are as follows: *β*-Thal major, *β*-Thal intermedia, and *β*-Thal trait [11,12,13]. *β*-Thal major and *β*-Thal intermedia are characterized by an elevated fetal hemoglobin percentage (HbF%.) They are associated with severe, moderate, or mild anemia requiring repeated transfusions resulting in a risk of iron overload. *β*-Thal trait is characterized by a high HbA% along with increased levels of hemoglobin A_2_ (HbA_2_% > 3.5%) and is associated with mild or no anemia and with variable microcytosis [14]. The current centralized test used for screening and diagnosing *β*-Thal is high-performance liquid chromatography (HPLC). This test relies on often unaffordable (USD 15 k–35 k or GHS 90 k–210 k) specialized instruments, state-of-the-art laboratory facilities, and highly trained personnel, which are lacking in the low-resource settings where *β*-Thal is most prevalent [2]. As a result, there is a need for affordable, portable, easy-to-use, and accurate point-of-care (POC) tests to facilitate decentralized *β*-Thal testing in low-resource settings [15,16,17,18].

**Figure 1:**
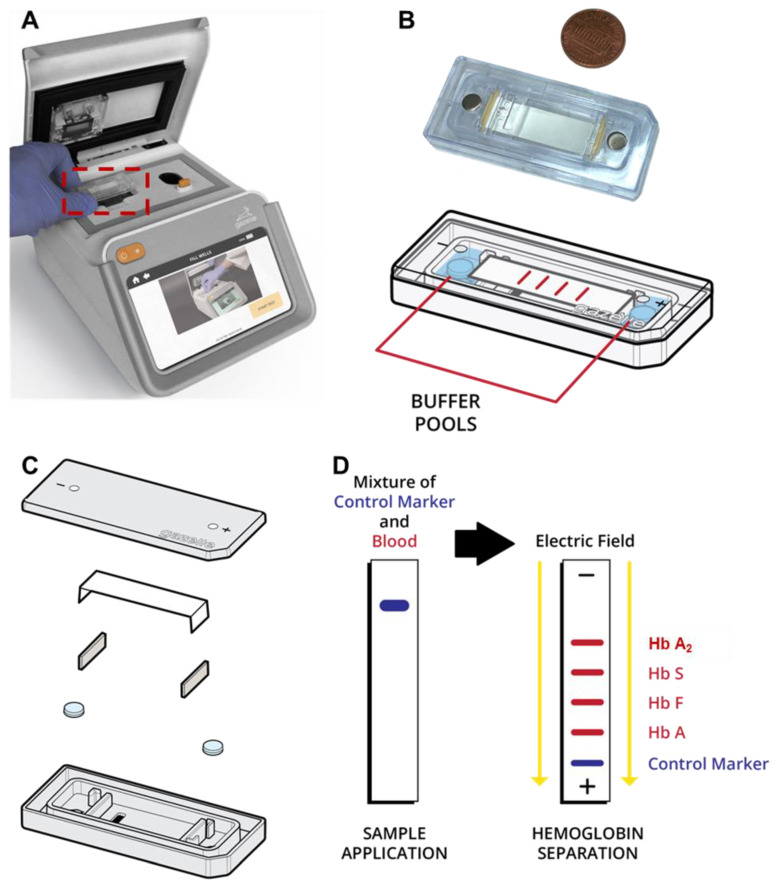
Gazelle paper-based microchip electrophoresis. (**A**) Gazelle paper-based microchip electrophoresis using a disposable cartridge (red boxed area, (**B**)) at the point of need. (**C**) The microcartridge is composed of a cartridge top, cellulose acetate paper, two blotting pads, a pair of stainless steel electrodes, and a cartridge bottom. (**D**) A detailed schematic illustrating the separation process of hemoglobin variants using the Gazelle system. Initially, lysed whole blood (red color), marked with a control blue dye (blue color) for visualization, is applied to the cellulose acetate paper housed within the microcartridge. Following this, an electric field is applied, thereby facilitating the separation of different hemoglobin variants based on their distinct electrophoretic mobilities.

Several POC diagnostic systems for several hemoglobin variants such as Hb S have been described [15,16,17,18,19,20,21] based on testing methods such as the sickle cell solubility test and antibody-based lateral flow assays such as Sickle SCAN^TM^ and HemoType SC^TM^ [22,23,24]. However, there is currently no POC test available for *β*-Thal detection. In a 2019 report, the World Health Organization (WHO) listed hemoglobin testing as one of the most essential for in vitro diagnostic (IVD) tests for primary care use in low- and middle-income countries [25,26]. Furthermore, hemoglobin electrophoresis has recently been added to the WHO’s essential list of IVDs for diagnosing sickle cell disease (SCD) and sickle cell trait [27]. Leveraging the WHO-recognized Hb electrophoresis test, we developed a paper-based, affordable (~$2/test and <$1000 for Gazelle^TM^ reader), and miniaturized Hb electrophoresis platform: Gazelle^TM^ (Figure 1) [28,29,30]. Gazelle^TM^ leverages an injection-molded, single-use, plastic microcartridge that can be mass-produced. This microcartridge embodies a pair of biomedical-grade stainless steel electrodes, a pair of blotting pads for maintaining current continuity, and cellulose acetate paper as a separation medium for electrophoresis (Figure 1B,C).

The fundamental principle behind Gazelle^TM^ is hemoglobin electrophoresis in which different hemoglobin variants can be separated based on mobility differences under an electric field (Figure 1D). Gazelle has been tested in clinical studies in four different countries with more than 700 subjects and demonstrated a capability of identifying major Hb variants, including HbA, HbE, HbS, and HbF, in adults as well as in newborns with SCD, sickle cell trait, hemoglobin C disorder, and hemoglobin E disorder [28,30,31,32].

Here, we implemented a customized image analysis algorithm for the accurate quantification of HbA_2_ for the first time in addition to HbA, HbF, and Hb C/E. Additionally, we described a test for evaluating the diagnostic performance of this platform in identifying *β*-Thal major, *β*-Thal intermedia, and *β*-Thal trait across 372 subjects in the age range of 4–63 years at Apple Diagnostics lab in Ghatkopar, Mumbai, India. Additionally, 32 blood samples were prepared to mimic *β*-thalassemia intermediate and *β*-thalassemia major samples. Gazelle-detected levels of Hb A, Hb F, and Hb A_2_ demonstrated high correlations with the results reported through laboratory gold standard high-performance liquid chromatography (HPLC) yielding a Pearson correlation coefficient of 0.99. These results suggest that Gazelle-Multispectral is potentially suitable for large-scale *β*-Thal testing.

## 2. Methods

### 2.1. Study Design and Oversight

We conducted a prospective diagnostic accuracy study on the use of Gazelle in detecting Hb variants including HbA, Hb, and HbA_2_ at Apple Diagnostics lab in Ghatkopar, Mumbai, India, adhering to an IRB-approved study protocol (IEC@IISS (International Institute of Sleep Sciences IRB number); Reg No.: ECR/177/Indt/MH-2014/RR-19). The results obtained from Gazelle were compared with the results reported through the reference (“Gold-standard [33]”) tests using HPLC in India. All samples were tested within 7 days of collection. All authors have reviewed and analyzed the data and attest to their accuracy and completeness as well as the fidelity of adherence to the study protocol.

### 2.2. Study Populations and Procedures

This test was conducted at Apple Diagnostics in Mumbai, India. Blood samples were collected from subjects at screening camps and education and awareness programs conducted by Plasma Labs in Jalgaon district, Maharashtra, India. All these subjects had consented to participate in the study. Subjects were excluded only if they had undergone a blood transfusion in the preceding 3 months or if they were unable to give informed consent. The study was approved by the IRB of the International Institute of Sleep Sciences (IISS), and informed consent was obtained from each participant’s parent or guardian if required. All the blood samples were collected on-site in EDTA blood collection tubes and stored at 4 C until they were tested through Gazelle. Blood samples were tested within one week of collection. The leftover blood was stored at 4C for HPLC testing using a D-10 HPLC system (Bio-Rad Laboratories, Hercules, CA, USA). The laboratory technician who conducted the tests through Gazelle had basic laboratory skills, such as pipetting and vortexing, and was able to independently perform the tests with less than 2 h of training.

### 2.3. Gazelle Test Procedure

The Gazelle^TM^ system design, including the microcartridge structure, paper-based electrophoresis principle, instrument design, and optical path structure, can be found in our previous publications [30,32]. The technicians performed the tests according to the Gazelle-Multispectral instructions for use as published previously [30]. Similarly, all buffer compositions have been published previously [30]. At the conclusion of the 8 min test, Gazelle automatically reports the percentages of each hemoglobin type present in the blood samples as well as an interpretative statement.

### 2.4. Confirmatory Laboratory Procedures

Samples that were stored at 4 °C degrees were retrieved. A total of 5 microliters of the sample were pipetted and diluted using 1500 microliters of distilled water. Diluted hemolysates were arranged on racks and loaded into a Bio-Rad D-10 HPLC system. The barcoded sample IDs were then edited to the match sample IDs. Each sample was run for approximately 6 min.

### 2.5. Gazelle-Multispectral Image Acquisition and Data Analysis

A customized image acquisition system and data analysis algorithm are integrated into the Gazelle system. The image acquisition system illuminates the microcartridge using a 410 nm wavelength ultra-violet (UV) LED. The imaging camera is positioned on the opposite side of the microcartridge from the UV light source and records electrophoresis progression at a rate of 0.25 frames per second. The Gazelle^TM^ image acquisition system allows us to acquire real-time images to track the separation and migration of hemoglobin bands on the cellulose acetate paper within the microcartridge (Figure 1B–D).

**Figure 2:**
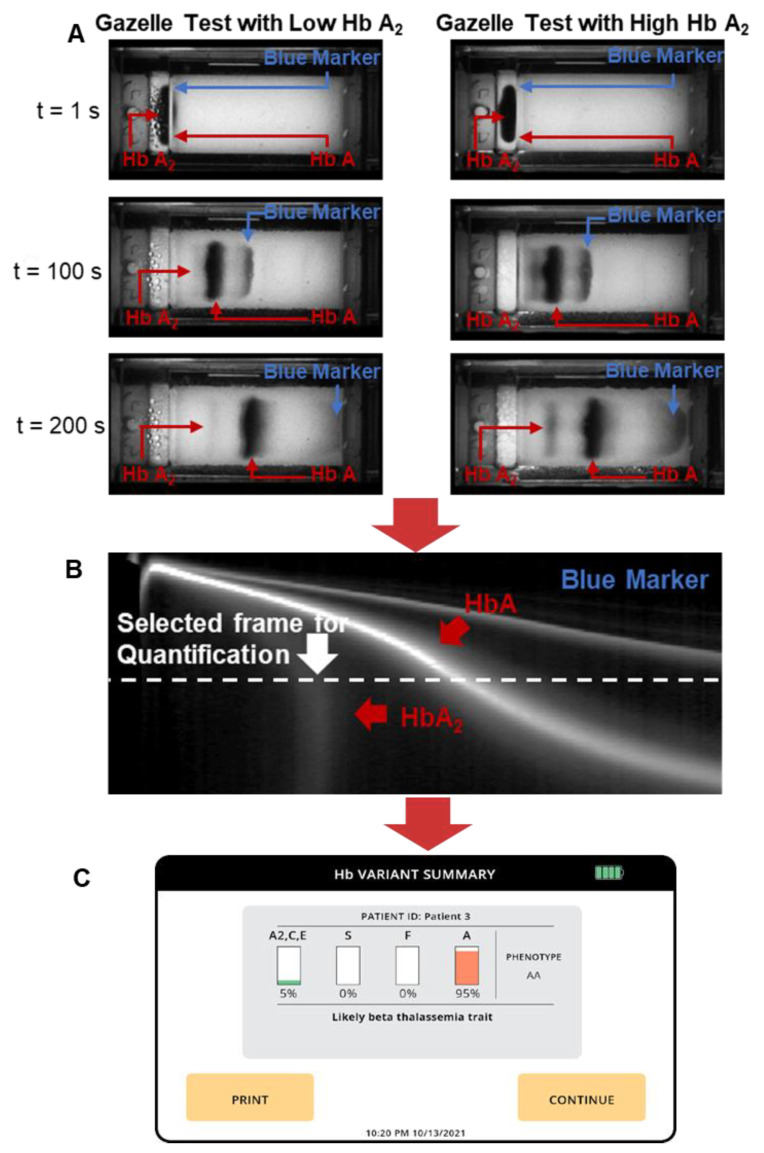
Gazelle tests for screening beta-thalassemia. (**A**) GazelleTM allows for real-time image acquisition for tracking hemoglobin migration and separation. Left: example of a Gazelle test of a blood sample with a low Hb A2 level. Right: example of a Gazelle test of a blood sample with a high Hb A2 level. (**B**) Applying an internally integrated data analysis algorithm, the generated space-time plots based on the captured images are used for the identification and quantification of Hb variants in real time. (**C**) At the end of each test, the Gazelle algorithm automatically reports the identified and quantified Hb variant results as well as the patient phenotype.

The Gazelle^TM^ data analysis algorithm automatically identifies *β*-Thal major, *β*-Thal intermedia, and *β*-Thal trait based on the Hb band migration pattern as described previously [30,34]. To put it briefly, the images acquired under 410 nm illumination are used to construct a space-time plot demonstrating the entire band electromigration process during each electrophoresis test (Figure 2B, *x*-axis: distance along the *x*-axis of the cellulose acetate paper; y-axis: time (from 0 to 480 s from top to bottom). Each point on the *x*-axis contains an averaged pixel intensity along the *y*-axis of the cellulose paper. *β*-thal detection requires the accurate quantification of HbA_2_. Our previously developed algorithms allowed for the detection and quantification of HbS, HbA, and HbF but not of HbA_2_; therefore, they are not suited for *β*-thal detection. Here, we developed a new algorithm for HbA_2_ quantification. This new algorithm includes two steps. In step one, the algorithm implements a matched filter in which it searches for the maximum signal-to-noise ratio of the imaged Hb band within a dynamic area in the space-time diagram (Figure 2B). The dynamic region is defined by two criteria used to track the sample loading position and HbA migration. Criterion one defines the time period (*y*-axis on the space-time diagram) within which the matched filter searches. This criterion is defined by how it starts upon 3–5 frames (0.25 fps, 12–20 s) after the HbA band and how it ends upon the 85th frame (340 s). Criterion two defines the position of the band along the length of the cellulose acetate paper (*x*-axis on the space-time diagram). This criterion is defined between column 73 (2.86 mm) and column 220 (8.63 mm) after the sample loading location. If the HbA_2_ signal is not identified in step one, then the algorithm stops. When HbA_2_ signals are identified in step one, the algorithm moves to step one. In step two, the algorithm calculates the amplitude of the HbA_2_ peak and the area underneath it using the electropherogram. The peak amplitude and area, along with the amplitudes of the non-A_2_ Hb variant peaks, are input into a regression algorithm that has been previously trained using HbA_2_ levels reported through HPLC. The algorithm then provides a quantitative estimation of HbA_2_. If the estimated HbA_2_ is above 3.5%, the algorithm will report a positive detection result for *β*-thal. At the end of each test, the Gazelle algorithm automatically reports the identified and quantified Hb variant results as well as the patient phenotype (Figure 2C).

**Figure 3:**
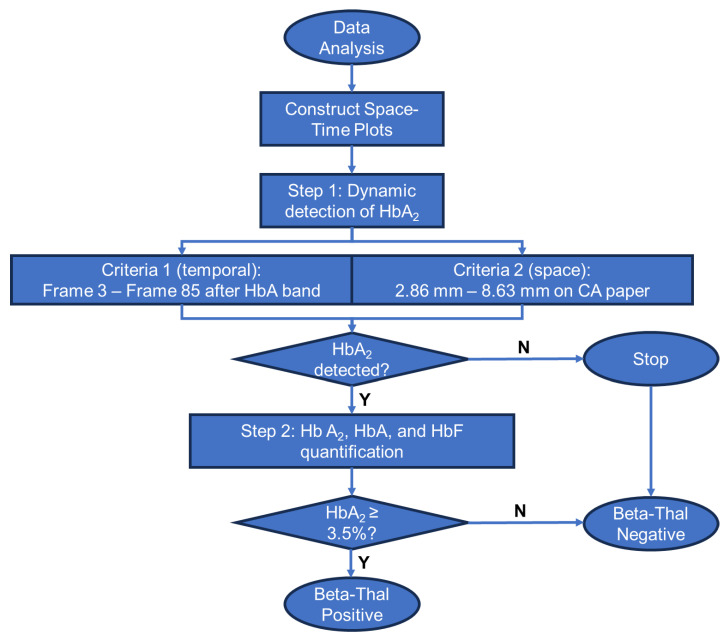
Process flow diagram of the Gazelle^TM^ data analysis algorithm for beta-thalassemia detection and quantification.

In addition to HbA_2_, the data analysis algorithm also automatically quantifies the relative percentages of HbA and HbF alongside other types of hemoglobin as previously reported. Gazelle’s reported results on Hb variant identification and quantification were compared with the ones reported through HPLC using Pearson correlation and Bland–Altman analysis. Gazelle-Multispectral sensitivity and specificity in the identification of *β*-Thal major, *β*-Thal intermedia, and *β*-Thal trait were calculated for the study population compared with the HPLC-reported results (Figure 3).

## 3. Results 

### 3.1. Test Population and Result Reporting

In this study, we conducted clinical testing across 372 subjects in the age range of 4–63 years at Apple Diagnostics lab in Ghatkopar, Mumbai, India, adhering to the local IRB-approved study protocol. Additionally, 32 blood samples were artificially prepared to mimic *β*-Thal major and *β*-Thal intermedia samples.

The Gazelle algorithm verifies the quality of test results according to the internally embedded data quality control (QC) method. According to the QC method, Gazelle-Multispectral organizes test results under one of the three categories of (1) “valid” test; (2) “uninterpretable” test; and (3) “inconclusive” test. The “valid”, “uninterpretable”, and “inconclusive” tests were defined according to the published recommendations in a previous publication [35] and the STARD guidelines [36]. If a test is performed as expected according to objective standards, the data analysis algorithm recognizes the test as a “valid” test and reports the test result. If a test includes poor migration of the blue control marker, an electrical connectivity issue, or faulty cartridges, the data analysis algorithm recognizes the test as an “uninterpretable” test. If a test is performed adequately according to an objective set of standards but has a quantification confidence value that is lower than the preset threshold value, then the data analysis algorithm recognizes the test as an “inconclusive” test. Reasons for “inconclusive” tests include the appearance of a Hb variant band or bands at or close to the borderline region between two adjacent detection windows. In this study, 394 out of 404 (97.5%) tests were recognized as “valid”, while 10 out of 404 (2.5%) tests were recognized as “inconclusive”.

### 3.2. Gazelle Result Interpretation Criteria

The Gazelle result interpretation criteria were established according to the clinical recommendations from key opinion leaders and using a large training dataset collected from field studies. The Gazelle *β*-Thal algorithm defines *β*-Thal major, *β*-Thal intermedia, and *β*-Thal trait according to the detected HbA, HbF, and HbA_2_ levels. The algorithm reads and interprets the data in the following sequences: (1) First, the algorithm checks if HbF ≥ 60% and HbA < 40% with no other hemoglobins over 10%. If true, the algorithm calls the sample *β*-Thal major or *β*-Thal intermedia. (2) When HbF < 60% and HbA ≥ 40% with no other hemoglobins over 10%, the algorithm measures the HbA_2_ level and then calls the sample *β*-Thal trait if HbA_2_ > 3.5%.

### 3.3. Gazelle-Multispectral Hb Variant Quantification Demonstrated a High Correlation with HPLC

**Figure 4:**
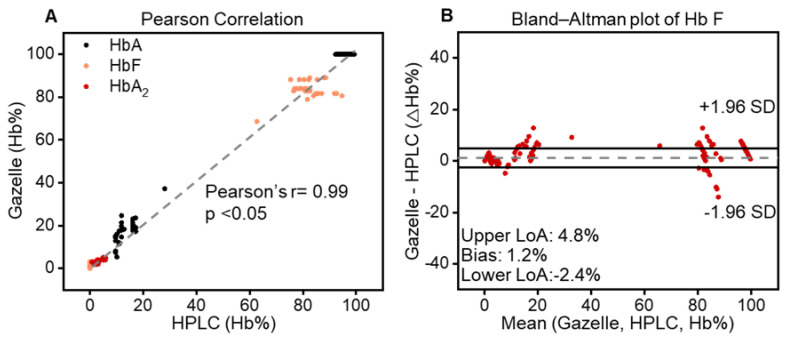
Gazelle accurately identifies and quantifies HbA, HbF, and HbA_2_. (**A**) Pearson correlation showed that Gazelle identified and quantified HbA (black), HbF (orange), and HbA_2_ (red), which demonstrated a strong correlation with the results reported through laboratory gold standard HPLC testing [33] at a Person coefficient correlation (PCC) of 0.99, *p* < 0.05). (**B**) Bland–Altman analysis demonstrated that Gazelle tests had an overall mean bias of 1.2% (doted gray line), with upper and lower limits of agreement (LoA) at 4.8% and −2.4%, respectively).

Pearson correlation analysis and Bland–Altman analysis were performed on the 394 tests recognized as “valid”. The correlation plots include the Gazelle-determined Hb variant levels (*y*-axis) versus the Hb variant levels reported through the HPLC (*x*-axis), including HbA (black), HbF (orange), and HbA_2_ (red, Figure 4A). The Bland–Altman analysis includes the difference between the Gazelle-quantified levels of HbA, HbF, and HbA_2_ and the ones determined through HPLC (*y*-axis) over the entire range of Hb levels detected (*x*-axis, Figure 4B). The result from the Pearson correlation analysis was a Pearson correlation coefficient (PCC) of 0.99 for all three Hb variants. Bland–Altman analysis results indicate that Gazelle determines blood Hb variant levels with a mean bias of +1.2%, with upper and lower limits of agreement at 4.8% and −2.4%, respectively. Taken together, these results demonstrated a good agreement between the Hb variant levels determined using Gazelle and the ones reported through HPLC (Figure 4).

### 3.4. Sensitivity and Specificity of Gazelle β-Thalassemia Testing

In this clinical study, Gazelle test results included disease (*β*-Thal major and *β*-Thal intermedia), trait (*β*-Thal trait), and normal (categorized using data interpretation criteria described above). Comparing the results reported from the laboratory standard test HPLC, Gazelle identified subjects with the disease, *β*-Thal major and *β*-Thal intermedia, from normal subjects and subjects with the *β*-Thal trait with 100% sensitivity and specificity (Table 1)**,** 6 normal subjects were identified as *β*-Thal trait. Sensitivity and specificity for identifying subjects with *β*-Thal trait from normal subjects (Trait vs. Normal) were 100% and 98.3%, respectively.

## 4. Conclusions

*β*-Thal major and *β*-Thal intermedia are characterized by an elevated fetal hemoglobin percentage (HbF%), and *β*-Thal trait is characterized by a high HbA% with increased levels of HbA_2_% [14]. Proper and timely management of *β*-Thal relies on early and accurate detection. The quantification of HbA and key Hb variants, including HbF and HbA_2_, are essential for the accurate detection of *β*-Thal. The previous Gazelle system has demonstrated its utility in detecting anemia [34] and hemoglobinopathies including SCD, sickle cell trait, Hemoglobin C disorder, and Hemoglobin E Disorder [28,29,30]. Here, we report the updated version of Gazelle that enables, for the first time, POC detection of *β*-Thal.

In this clinical study conducted among 404 subjects in Ghatkopar, Mumbai, India, Gazelle demonstrated 100% sensitivity and specificity for identifying *β*-Thal major/intermedia vs. healthy subjects as well as subjects with *β*-Thal major/intermedia vs. *β*-Thal trait. Additionally, Gazelle demonstrated 100% sensitivity and 98.3% specificity for identifying *β*-Thal trait vs. healthy subjects. A recommended practice in Hb testing is that all positive test results are confirmed through a secondary method prior to final diagnostic decision making and treatment initiation [37]. Therefore, all disease-positive tests would likely result in a secondary confirmatory test that should eliminate potential false positives if there are any.

In summary, Gazelle provides an affordable and rapid POC solution for detecting *β*-Thal for the first time. The Gazelle reader provides (1) animated on-screen instructions with step-by-step guidance for test operation procedures to minimize user errors and (2) a data analysis algorithm that automatically reports Hb variant levels and the predicted disease status. Overall, the results reported in this study suggest that Gazelle is potentially suitable for large-scale *β*-Thal testing in low-resource regions where the prevalence of *β*-Thal is high.

## Figures and Tables

**Table 1:** Gazelle-Multispectral screening sensitivity, specificity, positive predictive value (PPV), and negative predictive value (NPV) in comparison with reference standard method ^a^.

	Disease vs. Normal Normal	Disease vs. Trait	Trait vs. Normal
True positive, TP	32	32	18
True negative, TN	344	18	338
False Positive, FP	0	0	6 ^b^
False negative, FN	0	0	0
Sensitivity, TP/(TP + FN)	100.0%	100.0%	100%
Specificity, TN/(TN + FP)	100.0%	100.0%	98.3%

^a^ 394 “valid” tests. Seven tests were recognized as “inconclusive” due to a failed quality control test; two tests were recognized as “inconclusive” due to an unknown result reported through HPLC; and one test was recognized as “inconclusive” due to an unknown result reported through Gazelle. “Inconclusive” tests did not generate a result that could be included in the sensitivity and specificity analysis [35,36]. ^b^ 6 normal subjects with HbA were recognized as having *β*-Thal trait.

## Data Availability

All reasonable requests for materials and data will be fulfilled by the corresponding author of this publication.

## References

[1] WHO (2022). Newborn Health in the Western Pacific. https://www.who.int/westernpacific/health-topics/newborn-health.

[2] Taher A.T., Musallam K.M., Cappellini M.D. (2021). *β*-Thalassemias. N. Engl. J. Med..

[3] Modell B., Darlison M. (2008). Global epidemiology of haemoglobin disorders and derived service indicators. Bull. World Health Organ..

[4] Somervaille T. (2001). Disorders of Hemoglobin: Genetics, Pathophysiology, and Clinical Management. J. R. Soc. Med..

[5] Bender M.A., Hulihan M., Dorley M.C., del Pilar Aguinaga M., Ojodu J., Yusuf C. (2021). Newborn Screening Practices for Beta-Thalassemia in the United States. Int. J. Neonatal Screen..

[6] Cao A. (2002). Carrier screening and genetic counselling in beta-thalassemia. Int. J. Hematol..

[7] Bhattacharya S., Thiyagarajan A., Sharma N., Srivastava A., Dhar D.K. (2019). Need for a universal thalassemia screening programme in India?. A public health perspective. J. Fam. Med. Prim. Care.

[8] Taher A.T., Bou-Fakhredin R., Kattamis A., Viprakasit V., Cappellini M.D. (2021). Improving outcomes and quality of life for patients with transfusion-dependent *β*-thalassemia: Recommendations for best clinical practice and the use of novel treatment strategies. Expert Rev. Hematol..

[9] Arif F., Fayyaz J., Hamid A. (2008). Awareness among parents of children with thalassemia major. J. Pak. Med. Assoc..

[10] Kattamis A., Forni G.L., Aydinok Y., Viprakasit V. (2020). Changing patterns in the epidemiology of *β*-thalassemia. Eur. J. Haematol..

[11] Danjou F., Anni F., Galanello R. (2011). Beta-thalassemia: From genotype to phenotype. Haematologica.

[12] Origa R. (1993). Beta-Thalassemia.

[13] Origa R. (2017). *β*-Thalassemia. Genet. Med..

[14] Rund D., Rachmilewitz E. (2005). Beta-thalassemia. N. Engl. J. Med..

[15] Kawooya I., Kayongo E., Munube D., Mijumbi-Deve R., Elliott S., Vandermeer B., Sewankambo N. (2022). Point-of-care diagnostic tests for sickle cell disease. Cochrane Database Syst. Rev..

[16] Clemente F., Antonacci A., Giardi M.T., Frisulli V., Tambaro F.P., Scognamiglio V. (2023). Last Trends in Point-of-Care (POC) Diagnostics for the Management of Hematological Indices in Home Care Patients. Biosensors.

[17] Bond M., Hunt B., Flynn B., Huhtinen P., Ware R., Richards-Kortum R. (2017). Towards a point-of-care strip test to diagnose sickle cell anemia. PLoS ONE.

[18] McGann P.T., Hoppe C. (2017). The pressing need for point-of-care diagnostics for sickle cell disease: A review of current and future technologies. Blood Cells Mol. Dis..

[19] Arishi W.A., Alhadrami H.A., Zourob M. (2021). Techniques for the Detection of Sickle Cell Disease: A Review. Micromachines.

[20] Ilyas S., Simonson A.E., Asghar W. (2020). Emerging point-of-care technologies for sickle cell disease diagnostics. Clin. Chim. Acta.

[21] Jaja C., Edem-Hotah J., Shepherd J., Patel N., Xu H., Gibson R.W. (2020). Analytic Characteristics and Performance of Novel Immunoassay Point-of-Care Tests for Early Diagnosis of Sickle Cell Disease a Systematic Review. Point Care.

[22] Canning D.M., Huntsman R.G. (1970). An assessment of Sickledex as an alternative to the sickling test. J. Clin. Pathol..

[23] Steele C., Sinski A., Asibey J., Hardy-Dessources M.D., Elana G., Brennan C. (2019). Point-of-care screening for sickle cell disease in low-resource settings: A multi-center evaluation of HemoTypeSC, a novel rapid test. Am. J. Hematol..

[24] Kanter J., Telen M.J., Hoppe C., Roberts C.L., Kim J.S., Yang X. (2015). Validation of a novel point of care testing device for sickle cell disease. BMC Med..

[25] WHO World Health Organization (2019). First WHO Model List of Essential In Vitro Diagnostics.

[26] An R., Huang Y., Man Y., Valentine R.W., Kucukal E., Goreke U., Sekyonda Z., Piccone C., Owusu-Ansah A., Ahuja S. (2021). Emerging point-of-care technologies for anemia detection. Lab A Chip.

[27] WHO (2019). Second WHO Model List of Essential In Vitro Diagnostics.

[28] Qua K., Swiatkowski S.M., Gurkan U.A., Pelfrey C.M. (2021). A retrospective case study of successful translational research: Gazelle Hb variant point-of-care diagnostic device for sickle cell disease. J. Clin. Transl. Sci..

[29] An R., Hasan M.N., Man Y., Gurkan U.A. Integrated Point-of-Care Device for Anemia Detection and Hemoglobin Variant Identification. Proceedings of the 2019 IEEE Healthcare Innovations and Point of Care Technologies, (HI-POCT).

[30] Hasan M.N., Fraiwan A., An R., Alapan Y., Ung R., Akkus A., Xu J.Z., Rezac A.J., Kocmich N.J., Creary M.S. (2020). Paper-based microchip electrophoresis for point-of-care hemoglobin testing. Analyst.

[31] Hasan M.N., An R., Akkus A., Akkaynak D., Minerick A.R., Kharangate C.R., Gurkan U.A. (2021). Dynamic pH and Thermal Analysis of Paper-Based Microchip Electrophoresis. Micromachines.

[32] An R., Huang Y., Rocheleau A., Avanaki A., Thota P., Zhang Q., Man Y., Sekyonda Z., Segbefia C.I., Dei-Adomakoh Y. (2022). Multispectral imaging for MicroChip electrophoresis enables point-of-care newborn hemoglobin variant screening. Heliyon.

[33] Centers for Disease Control and Prevention (2015). Hemoglobinopathies: Current Practices for Screening, Confirmation and Follow-Up.

[34] An R., Man Y., Iram S., Kucukal E., Hasan M.N., Huang Y., Goreke U., Bode A., Hill A., Cheng K. (2021). Point-of-care microchip electrophoresis for integrated anemia and hemoglobin variant testing. Lab A Chip.

[35] Shinkins B., Thompson M., Mallett S., Perera R. (2013). Diagnostic accuracy studies: How to report and analyse inconclusive test results. BMJ Br. Med. J..

[36] Bossuyt P.M., Reitsma J.B., E Bruns D., A Gatsonis C., Glasziou P.P., Irwig L., Lijmer J.G., Moher D., Rennie D., de Vet H.C. (2015). STARD 2015: An Updated List of Essential Items for Reporting Diagnostic Accuracy Studies. Clin. Chem..

[37] Ryan K., Bain B.J., Worthington D., James J., Plews D., Mason A., Roper D., Rees D.C., De La Salle B., Streetly A. (2010). Significant haemoglobinopathies: Guidelines for screening and diagnosis. Br. J. Haematol..

